# Markers associated with genomic instability, immunogenicity and immune therapy responsiveness in Metaplastic carcinoma of the breast: Expression of γH2AX, pRPA2, P53, PD-L1 and tumor infiltrating lymphocytes in 76 cases

**DOI:** 10.1186/s12885-022-10408-7

**Published:** 2022-12-12

**Authors:** S. Voutilainen, P. Heikkilä, J. Bartkova, H. Nevanlinna, C. Blomqvist, J. Bartek, J. Mattson

**Affiliations:** 1 Helsinki University Hospital Comprehensive Cancer Centre, Paciuksenkatu 3, PO BOX 180, 00290 Helsinki, Finland; 2 Department of Pathology, University of Helsinki and HUSLAB, Helsinki University Hospital, Helsinki, Finland; 3 Danish Cancer Society Research Center, Copenhagen, Denmark; 4 Division of Genome Biology, Department of Medical Biochemistry and Biophysics, Science for Life Laboratory, Karolinska Institute, Stockholm, Sweden; 5 Department of Obstetrics and Gynecology, University of Helsinki, Helsinki University Hospital, Helsinki, Finland

**Keywords:** Metaplastic breast cancer, PD-L1, p53, γH2AX, TILs, RPA2

## Abstract

**Background:**

Metaplastic breast cancer (MpBC) is an aggressive subtype of breast carcinoma that is often resistant to conventional chemotherapy. Therefore, novel treatment strategies are urgently needed. Immune check point inhibitors have shown activity in programmed death-ligand 1 (PD-L1) – positive metastatic triple negative breast carcinoma (TNBC), which raises the possibility that immunotherapy may also be effective in MpBC as most of the MpBCs are triple negative. The aim of the present study was to assess genomic instability and immunogenicity in tumor specimens of patients with MpBC.

**Methods:**

A total of 76 patients diagnosed with MpBC over a 15-year period were included in the study. We performed immunohistochemical analyses for tumor cell PD-L1, immune cell PD-L1 and p53 on tissue microarrays (TMAs), analyzed stromal and intratumoral tumor infiltrating lymphocytes (TILs) from hematoxylin and eosin-stained (H&E) slides and scored gamma-H2AX (γH2AX) and phosphorylated-RPA2 (pRPA2) from whole tissue sections. We correlated marker expression with clinicopathologic features and clinical outcome.

**Results:**

All tumors expressed γH2AX and pRPA2 with median expressions of 43% and 44%. P53- (68%), tumor cell PD-L1- (59%) and immune cell PD-L1-positivity (62%) were common in MpBCs. Median stromal TIL and intratumoral TIL counts were 5% and 0. The spindle and squamous cell carcinomas expressed the highest levels of PD-L1 and TILs, and carcinoma with mesenchymal differentiation the lowest.

**Conclusions:**

MpBC appears to be an immunogenic cancer with high genomic instability and frequent PD-L1-positivity, implying that check point inhibitors might be effective in MpBC. Expression levels of PD-L1 and TILs varied across different histologic subtypes, suggesting that immunotherapy might be less effective in carcinoma with mesenchymal differentiation.

**Supplementary Information:**

The online version contains supplementary material available at 10.1186/s12885-022-10408-7.

## Background

MpBC is a rare subtype of breast carcinoma but accounts for a significant proportion of breast cancer mortality. Patients with MpBC usually present with larger, higher grade and more often triple negative tumors compared with other patients with invasive ductal carcinoma. More importantly, MpBCs respond less frequently to chemotherapy and carry a worse prognosis compared with other TNBCs with a median overall survival less than one year in the metastatic setting [1, 2]. Due to its aggressiveness and poor response to chemotherapy, there is a great need for novel therapy targets.

Genomic instability is an important hallmark of cancer [3] and usually a marker of poor prognosis [4]. Only a few previous reports on genomic instability in MpBC have been published. The frequency of p53 mutations [5–7], and other pathogenic genomic alterations have been reported to be high, while the mutational burden has been highly variable, but in most cases low [7]. γH2AX is a widely used marker for double strand breaks and genomic instability[8–10]. Elevated levels of γH2AX are present in a number of human cancers [11]. In breast cancer γH2AX has been associated with BRCA1 and p53 mutations, triple negativity and the basal-like subgroup [12, 13]. The Replication protein A (RPA) is another marker of genomic instability and replication stress. RPA is required for each of the four major DNA repair pathways: nucleotide excision repair, base excision repair, DNA mismatch repair, and DNA double strand break -repair. RPA seems to facilitate tumor growth by helping cancer cells to withstand replication stress and is overexpressed and/or commonly phosphorylated by checkpoint kinases in various cancers [14, 15]. A significant upregulation of RPA2 has also been shown in breast cancer tissues and cell lines [16].

Tumor immunogenicity has been linked to genomic instability and a high tumor mutation burden (TMB) [17–19]. TMB is associated with both single strand (*p* < 0.001) and double strand DNA breaks (*p* < 0.001) [20] and to a higher probability of response to immunotherapy across different types of cancer [21, 22]. Although, TMB is significantly lower in breast cancer than in most other solid malignancies, other factors like TILs and PD-L1 may account for immune response in this disease [23]. Tumor infiltrating lymphocytes (TILs) are mononuclear immune cells that are found in stroma or within the tumor itself. TILs have prognostic value in TNBC and HER2-positive breast cancer and increased TIL concentration is associated with improved DFS and OS in patients treated with either adjuvant or neoadjuvant therapy [24, 25]. Programmed cell death protein 1 (PD-1) is a cell surface receptor that is commonly found on T-cells and acts to block T-cell activation. PD-L1, a ligand for PD-1, is expressed in a variety of cancers and its binding to PD-1 leads to inactivation of TILs, and helps cancer cells to escape antitumor immune response [26]. PDL-1 inhibitor atezolizumab increased overall survival by seven months in the first line treatment of metastatic TNBC in the subgroup of patients with PDL1 expression [27].

The aim of the present study was to examine genomic instability by analyzing p53, γH2AX and pRPA2 and immunogenicity by analyzing TILs and PD-L1 in tumor specimens of patients with MpBC.

## Methods

### Patients and samples

The study was approved by the Ethics committee of the Helsinki University Hospital. Since this was a retrospective archive study consent from the participants was not required. The pathology databases were searched over a 15-year period (2002—2016) for patients with a histologically confirmed diagnosis of metaplastic carcinoma who were treated at the Helsinki University Hospital Comprehensive Cancer Centre. All cases were reviewed by a breast pathologist in order to verify the diagnosis and histological subtype classification. Tumor tissue was subclassified according to the WHO Classification of Tumours of the Breast into low-grade adenosquamous carcinoma, fibromatosis-like metaplastic carcinoma, squamous cell carcinoma, spindle cell carcinoma and carcinoma with mesenchymal differentiation [28]. MpBCs having different metaplastic components were classified as mixed metaplastic carcinomas and MpBCs associated with conventional breast cancer components as mixed type.

A total of 76 patients fulfilled the criteria and were included in this study. Stromal and intratumoral TILs, γH2AX and pRPA2 were evaluated from whole tissue sections. PD-L1 and P53 were predominantly assessed from TMAs. However, whole tissue sections were used if the cores were dislodged or failed to contain tumor tissue. Due to inadequate material in three patients, seventy-five patients were included in the analysis of γH2AX, 74 in P53 and RPA2 and 73 in PD-L1.

Ki-67 (MIB-1), estrogen (ER) and progesterone receptor (PR) and HER2 scores were collected from routine diagnostic reports. Ki-67 was considered high if ≥ 20% of cells stained positively at hot spots, and ER and PR as positive if ≥ 10% of cells stained positively. We chose to define the basal-like subtype as either EGFR or CK5/6 –positivity irrespective of ER-status [29].

The following equipment was used for the microscopic images: microscope (Nikon Eclipse Ci); objective lenses (Nikon Plan Fluor); camera (Leica MC 190 HD); software (Leica Application Suite LAS EZ Version 3.4.0 (Build:272)).

### Immunohistochemistry and diagnostics for TILs

γH2AX and pRPA2 indirect immunoperoxidase staining was carried out using formaldehyde-fixed, paraffin embedded tumor sections. For antigen unmasking, the deparaffinized sections were boiled in a microwave oven for 15- 20 min in citrate buffer (pH = 6), followed by overnight incubation with the primary rabbit antibody against RPA2 phosphorylated at Ser 4 and 8 (pRPA2) from Novus Biologicals (γH2AX, dilution 1:1500), or the mouse monoclonal antibody to human histone H2A.X phosphorylated at Ser 139 (Millipore, clone JBW301, diluted 1:2500). As secondary reagents and the enhanced chromogen reaction, we then used the Vectastain Elite Kit (Vector Laboratories Burlingame, CA, USA) and nickel—sulphate—enhancement step without nuclear counterstaining to visualize the chromogenic (diaminobenzidine) reaction. As negative controls, sections were incubated with nonimmune rabbit or mouse sera, respectively, and human glioblastoma sections served as positive control with pronounced γH2AX and pRPA2 signal. The evaluation of staining was performed by an experienced pathologist, by counting percentage of positive tumor cells in areas of each section corresponding to the most positive 25% of each evaluated section [8, 30].

For the assessment of p53, tumor cell PD-L1 and immune cell PD-L1, H&E slides were reviewed and representative areas were marked out on the matching formalin-fixed, paraffin embedded tissue blocks. Either one- (when tumor tissue was scarce) or two-millimeter cores from paraffin blocks were used to construct TMAs (four cores per case). Overexpression of p53 (Dako, M7001, 1:500, Ventana) was defined as nuclear staining in ≥ 10% of the tumor cells [5, 31–33]. PD-L1 was evaluated by using the VENTANA SP142 assay. Scoring of PD-L1 expressing immune cells and tumor cells was based on the percentage of tumor area and tumor cells, respectively. Positive staining was defined as ≥ 1% of cells with any intensity [34].

Stromal and intratumoral TILs were evaluated on H&E stained sections following the TILs working group recommendations and expressed as percentages [35]. Lymphocyte-predominant breast cancer (LPBC) was classified as either a stromal or intratumoral TIL count equal or exceeding 50%.

### Statistical analysis

For the association of biomarkers to clinicopathologic characteristics and their correlation to each other either a Spearman rank correlation coefficient or a Mann–Whitney U-test was used. The analyses of P53 overexpression were done with a Mann Whitney U-test or with Fisher’s exact test / χ2 test. The association between markers and histological subtype was tested with the Kruskal–Wallis test for continuous variables and χ2 test for dichotomous P53. All the tests were 2-sided. Cox Regression analysis was performed to evaluate the prognostic impact of these biomarkers on disease free survival (DFS) and breast cancer specific overall survival (BCOS). Patients with metastasis at diagnoses (*n* = 2) were excluded from the analysis of DFS and BCOS. A *p*-value < 0.01 was considered statistically significant instead of the conventional value of 0.05 due to the problem of multiple tests. The analyses were performed with SPSS statistical package version 25.

## Results

### Markers of genomic instability

**Fig. 1:**
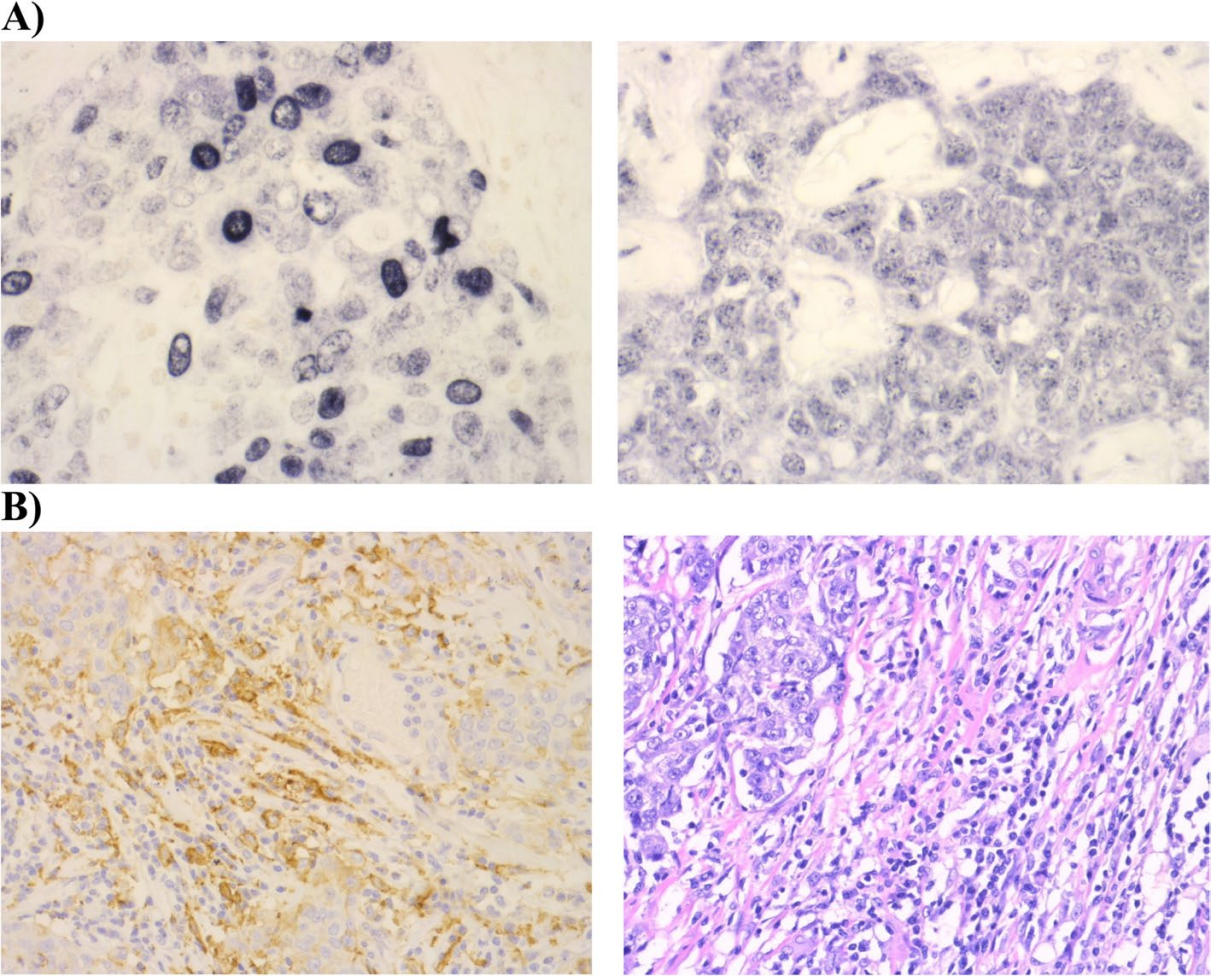
Representative images of immunohistochemical staining of γH2AX, pRPA2, PD-L1 and TILs. **A** Representative examples of heterogenous staining patterns for γH2AX (left) and phospho-RPA (right) in breast carcinoma; magnification: 40 × lens. **B** Representative examples of PD-L1 positive lymphocytes and tumor cells (left) and tumor infiltrating lymphocytes in HE-stained section (right); magnification: 20 × lens

P53 expression was positive in 68% (50/74) of the tumors. All cases expressed both γH2AX and RPA2, indicating that these tumors have an aberrant replication stress and DNA damage signaling. The mean and median expressions for γH2AX were 46% of the tumor cells stained positively and 43% of the tumor cells stained positively (*n* = 75, range 5—85) and for pRPA2 42% of the tumor cells stained positively and 44% of the tumor cells stained positively (*n* = 74, range 3 – 81). There was a highly significant statistical correlation (*p* < 0.0005) between RPA2 and γH2AX (Table 1). Representative images of staining patterns for γH2AX and pRPA2 are shown in Fig. 1 A. In this tumor specimen the expressions of γH2AX and pRPA2 were 45% and 65%.

**Table 1:** Correlation coefficients of immunohistochemical biomarkers

	gH2AX	RPA2	tcPD-L1	icPD-L1	sTIL
RPA2	0.530 (*p* < 0.0005)				
tcPD-L1	-0.045*	-0.085*			
icPD-L1	-0.157 (*p* = 0.034)	-0.124*	0.453 (*p* = 0.001)		
sTIL	0.051*	-0.059*	0.199*	0.453 (*p* < 0.0005)	
iTIL	0.094*	0.076*	0.295 (*p* = 0.007)	0.007*	0.041*

*sTIL* % stromal tumor-infiltrating lymphocytes, *iTIL* % intratumoral tumor-infiltrating lymphocytes, *tcPD-L1*% tumor cell-PD-L1, *icPD-L1*% immune cell PD-L1

^*^non-significant *p*-values

### Markers of immunogenicity

PD-L1 expression (≥ 1%) was detected in 59% (43/73) of the tumor cells and in 62% (45/73) of the immune stromal cells. Mean and median stromal TIL counts were 9% and 5% (*n* = 76, range 0 – 60) and mean and median intratumoral TIL counts were 2% and 0 (*n* = 76, range 0 – 20). Three cases (4%) were classified as LPBC. Two of these were of the squamous and one of the mixed subtypes. There were highly statistically significant positive correlations between immune cell PD-L1 and tumor cell PD-L1 (*p* = 0.001), immune cell PD-L1 and stromal TILs (*p* < 0.0005) and tumor cell PD-L1 and intratumoral TILs (*p* = 0.007). In addition, immune cell PD-L1 had a negative correlation with γH2AX (*p* = 0.034), although this finding did not reach the pre-set threshold of 0.01. Otherwise, immunologic markers did not correlate significantly with genomic markers (Table 1). Representative images of PD-L1 and TIL evaluation are shown in Fig. 1 B. In this case 60% of immune cells and 10% of tumor cells expressed PD-L1 and stromal and intratumoral TIL counts were 50% and 0, respectively.

### Association of markers of genomic instability and immunogenicity to clinicopathologic characteristics and histological subtypes

Clinicopathologic characteristics and immunohistochemical expression of biomarkers are summarized in Table 2. Analyses of the association of P53 and PD-L1-positivity with clinicopathologic characteristics can be found in the Supplementary table 1 (S1). The markers of genomic instability γH2AX and RPA2 did not correlate to any of the clinicopathological variables or histological subtypes. Tumor cell PD-L1 expression was associated with larger tumor size (*p* = 0.033) and intratumoral TIL in the basal phenotype (*p* = 0.048), although these p-values did not reach the pre-set threshold of 0.01. Stromal TIL was highly associated with HER2-positivity (*p* = 0.001).

**Table 2:** Clinicopathologic characteristics and immunohistochemical expression of biomarkers

Features	Groups (n)	gH2AX, % median (range) *n* = 75	*P*	RPA2, % median (range) *n* = 74	*P*	sTIL, % median (range) *n* = 76	*P*	iTIL, % median (range) *n* = 76	*P*	tcPD-L1,% median (range) *n* = 73	*P*	icPD-L1, % median (range) *n* = 73	*P*
Age at diagnosis^a,c^	≤ 50 (5) > 50 (71)	63 (30–85) 42 (5–85)	0.711	44 (20–73) 44 (3–81)	0.800	12 (5–30) 5 (0–60)	0.061	0 (0–5) 0 (0–20)	0.052	23 (0–100) 3 (0–100)	0.525	40 (5–60) 5 (0–80)	0.461
Grade^a^	1 (2) 2 (10) 3 (64)	70 (70–70) 42 (6–85) 43 (5–85)	0.860	20 (20–20) 52 (3–75) 44 (3–81)	0.985	5 (5–5) 2 (0–40) 5 (0–60)	0.404	0 (0–0) 1 (0–20) 0 (0–20)	0.907	0 (0–0) 1 (0–40) 5 (0–100)	0.138	16 (1–30) 0 (0–80) 20 (0–70)	0.248
Pathological tumor size (mm)^a,c^	≤ 20 (20) 21–50 (38) > 50 (17)	60 (8–85) 36 (6–85) 40 (5–79)	0.154	45 (0–60) 44 (3–81) 32 (4–80)	0.385	5 (0–60) 4 (0–40) 2 (0–50)	0.093	0 (0–20) 1 (0–20) 1 (0–5)	0.074	0 (0–80) 7 (0–100) 10 (0–80)	0.033	3 (0–60) 18 (0–80) 10 (0–60)	0.937
Pathological nodal stage^a^	N0 (57) N1 (7) N2 (2) N3 (3)	43 (6–85) 32 (5–83) 49 (19–79) 27 (11–28)	0.255	40 (3–80) 45 (4–65) 53 (25–80) 44 (38–75)	0.529	5 (0–50) 5 (0–50) 10 (0–20) 10 (7–25)	0.284	0 (0–20) 0 (0–2) 0 (0–0) 0 (0–2)	0.162	5 (0–100) 7 (0–80) 8 (6–10) 3 (0–15)	0.717	20 (0–80) 10 (0–60) 35 (0–70) 1 (0–15)	0.644
ER ^b^	> 10% (9) < 10% (67)	47 (8–80) 43 (5–85)	1.000	50 (7–81) 44 (3–80)	0.399	5 (0–40) 5 (0–60)	0.452	1 (0–20) 0 (0–20)	0.673	10 (0–80) 3 (0–100)	0.240	20 (0–80) 5 (0–70)	0.420
PR^b^	> 10% (2) < 10% (74)	42 (8–75) 43 (5–85)	0.833	45 (20–70) 44 (3–81)	0.831	4 (2–5) 5 (0–60)	0.909	0 (0–0) 0 (0–20)	0.295	0 (0–0) 3 (0–100)	0.171	30 (0–60) 10 (0–80)	0.804
HER2^b^	Positive (3) Negative (73)	67 (11–73) 43 (5–85)	0.889	69 (44–70) 43 (3–81)	0.199	50 (25–60) 5 (0–50)	0.001	2 (1–2) 0 (0–20)	0.222	1 (0–15) 3 (0–100)	0.743	1 (0–30) 10 (0–80)	0.743
Ki-67^b^	< 20% (9) ≥ 20% (67)	37 (6–85) 44 (5–85)	0.696	30 (3–75) 44 (3–81)	0.836	3 (0–40) 5 (0–60)	0.565	0 (0–20) 0 (0–20)	0.604	10 (0–100) 3 (0–100)	0.560	4 (0–80) 10 (0–70)	0.972
Basal phenotype^b^	Yes (13) No (61)	44 (5–85) 40 (8–83)	0.925	46 (3–81) 38 (3–80)	0.739	5 (0–60) 5 (0–30)	0.563	1 (0–20) 0 (0–20)	0.048	3 (0–100) 3 (0–80)	0.794	13 (0–80) 1 (0–60)	0.917

*sTIL* stromal tumor-infiltrating lymphocytes, *iTIL* intratumoral tumor-infiltrating lymphocytes, *tcPD-L1* tumor cell-PD-L1, *icPD-L1* immune cell PD-L1, ER estrogen receptor, *PR* progesterone receptor, HER2 human epidermal growth factor receptor 2

^a^Spearmann rank correlation coefficient

^b^Mann-Whitney

^c^Tested as Spearmann rank correlation coefficient with age and tumor size as continuous variables

Biomarker expression across different histological subtypes is summarized in Table 3. There was a statistically significant association between intratumoral TIL and histologic subtype (*p* < 0.0005). Also, tumor cell PD-L1 was associated with histological subtypes (*p* = 0.010). The median percentages of tumor cell PD-L1 were highest in spindle and squamous cell carcinomas and lowest in carcinoma with mesenchymal differentiation, whereas median intratumoral TIL counts were highest in spindle cell carcinoma and lowest in low grade adenosquamous carcinoma, carcinoma with mesenchymal differentiation and mixed type carcinomas.

**Table 3:** Expression of biomarkers across different histological subtypes

Subtype (n)	P53 ≥ 10% n (%) *n* = 74	tcPD-L1 median, (range) *n* = 73	icPD-L1 median, (range) *n* = 73	tcPD-L1 ≥ 1% n (%) *n* = 73	icPD-L1 ≥ 1% n (%) *n* = 73	γH2AX median, (range) *n* = 75	RPA2 median, (range) *n* = 74	iTIL median, (range) *n* = 76	sTIL median, (range) *n* = 76
Low grade adenosquamous (1)	0 (0)	0 (0–0)	30 (30–30)	0 (0)	1 (100)			0 (0–0)	5 (5–5)
Squamous (19)	15 (83)	13 (0 – 100)	20 (0 – 60)	11 (61)	13 (72)	43 (8 – 78)	40 (7 – 73)	1 (0 – 10)	7 (0 – 60)
Spindle (17)	12 (71)	30 (0 – 100)	5 (0 – 80)	13 (77)	12 (71)	43 (6 – 85)	40 (3 – 75)	3 (0 – 20)	3 (0 – 40)
Carcinoma with mesenchymal differentiation (13)	8 (62)	0 (0 – 5)	0 (0 – 40)	3 (25)	4 (33)	49 (15 – 83)	58 (12 – 81)	0 (0 – 2)	0 (0 – 40)
Mixed metaplastic (9)	6 (67)	7 (0 – 100)	20 (0 – 60)	7 (78)	7 (78)	49 (12 – 85)	25 (3 – 75)	1 (0 – 5)	5 (1 – 20)
Mixed type (17)	9 (56)	2 (0 – 90)	1 (0 – 70)	9 (56)	8 (50)	38 (5 – 79)	50 (4 – 80)	0 (0 – 2)	5 (0 – 50)
*P* value	0.375^b^	0.010^a^	0.368^a^			0.893^a^	0.271^a^	< 0.0005^a^	0.096^a^

*tcPD-L1* tumor cell-PD-L1, *icPD-L1* immune cell PD-L1, *iTIL* % intratumoral tumor-infiltrating lymphocytes, *sTIL* % stromal tumor-infiltrating lymphocytes

^a^Kruskal-Wallis test

^b^χ2 test

### Survival analyses

Cox Regression analyses for association of biomarkers with DFS and BCOS are shown in Table 4. There was an association of borderline significance between positive tumor cell PD-L1 expression and shorter DFS (HR 1.01, *p* = 0.032) and BCOS (HR 1.01, *p* = 0.035).

**Table 4:** Univariate Cox Regression analyses for association of biomarkers with clinical outcome

	Disease free Survival		Overall Survival	
Biomarkers	HR (95% CI)	*P* Value	HR (95% CI)	*P* Value
P53 ≥ 10%/ < 10%	1.30 (0.54 – 3.12)	0.554	1.57 (0.63 – 3.92)	0.339
tcPD-L1, %	1.01 (1.00 – 1.02)	0.032	1.01 (1.00 – 1.02)	0.035
icPD-L1, %	1.00 (0.98 – 1.02)	0.953	1.00 (0.99 – 1.02)	0.787
γH2AX, %	0.99 (0.97 – 1.01)	0.159	0.99 (0.98 – 1.01)	0.268
RPA2%	0.99 (0.98 – 1.01)	0.457	0.99 (0.97 – 1.01)	0.290
iTIL, %	1.03 (0.92 – 1.15)	0.627	1.03 (0.93 – 1.15)	0.534
sTIL, %	0.99 (0.96 – 1.03)	0.662	0.99 (0.96 – 1.02)	0.454

*HR* hazard ratio, *CI* confidence interval, *tcPD-L1* tumor cell PD-L1, *icPD-L1* immune cell PD-L1, *iTIL* intratumoral tumor-infiltrating lymphocytes, *sTIL* stromal tumor-infiltrating lymphocytes

## Discussion

Genomic instability and a high mutational burden have been associated with poor outcome of cancer patients not treated with immunotherapy [4]. However, a high mutational burden is also a predictive marker for positive clinical response to cancer immunotherapy [17–19]. In the present study we assessed overexpression of p53 and two markers of DNA damage/replication stress, γH2AX and pRPA2. We found p53-positivity in 68% of the tumors, which is in line with previous results of 61 – 71% in MpBC [5–7]. Lower rates of overexpression of p53 between 20 and 40% have generally been reported among unselected cases of invasive ductal carcinoma [5, 6]. To the best of our knowledge, there are no previous studies examining γH2AX- and pRPA2-expression in MpBC. Bartkova et al. examined the expression of γH2AX in breast cancer and reported a higher proportion of positivity (> 1%) in TNBC (67%, 86/129) than in tumors with positive hormone receptors or HER2 overexpression, (44%, 343/773) [13]. In the present study all MpBC cases were considered γH2AX positive in the same laboratory with similar methodology. A large breast cancer study by Yang et al. reported that 24% of the breast cancer tumors expressed γH2AX, and positivity was associated with higher tumor grade, triple negativity and p53-positivity [36]. In another breast cancer study (*n* = 110) the median expression of γH2AX was 45% and γH2AX was a marker of poor prognosis including large tumor size, high grade, number of metastatic lymph nodes, expression of HER2- and Ki-67, as well as ER- and PR-negativity (50). A Dutch study indicated that high expression of γH2AX was a poor prognostic factor in TNBC (*n* = 44) but not in the whole group of breast cancer (*n* = 122) [12]. Although, in previous studies with unspecified breast cancer patients there has been a link between p53-positivity and γH2AX [11–13], we could not find such association. Our results therefore suggest that besides the enhanced replication stress and DNA damage signaling, there might be also additional stress response pathways, the activity of which can create a tumor environment that favors selection of p53 mutations and/or p53 protein overabundance in human MpBC. γH2AX and pRPA2 were highly correlated but we found no statistically significant associations with other biomarkers or clinicopathologic characteristics. However, a previous study by Osoegawa et al. revealed a correlation between PD-L1 and γH2AX expression in 41 patients with pulmonary squamous cell carcinoma, suggesting that increased DNA damage may upregulate expression of PD-L1 and thereby sensitize such tumors to immunotherapy [37].

In the present study MpBC frequently expressed both immune cell PD-L1 (62%) and tumor cell PD-L1 (59%). According to a systematic review [38] PD-L1 expression in breast cancer has varied considerably, with rates ranging from 0 to 83% across subtypes and between 5 and 80% in TNBC. Most studies were based on TMA-technique and assessed the expression in either tumor cells alone or in both tumor and immune cells. In the largest single study (*n* = 3916) PD-L1-expression was analyzed from TMAs and reported separately for tumor and immune cells. With the cut-off of 1% for positivity, immune cell PD-L1-positivity was seen in only 6% of the tumors, but was considerably higher in TNBC (14%) and basal-like tumors (19%). The proportion of PD-L1-positivity in tumor cells were 1.7%, 6% and 9% in the whole cohort, TNBC and basal-like cancers, respectively [39]. The reported proportion of PD-L1-positivity has been considerably higher in patients with TNBC participating in recent immunotherapy trials. In IMpassion130 41% of patients were immune cell PD-L1-positive and 8.7% tumor cell PD-L1-positive [40], while the positivity rates (PD-L1 combined positive score ≥ 1) were 75% and 65% in the KEYNOTE-355 and the KEYNOTE-119 studies, respectively [41, 42]. Different methods and criteria for positivity, or the use of TMAs compared to whole tumor sections may at least partly explain the large variations in PD-L1 -expression, and complicates comparisons across studies.

A number of previous studies have studied PD-L1 expression in MpBC. In the largest of these a Taiwanese study by Lien et al*.* (*n* = 82) reported an overall positivity (≥ 1% expression) of 34% for immune cell PD-L1 and 17% for tumor cell PD-L1 [43]. In an American study (*n* = 75) comparing MpBC to other breast cancer subtypes PD-L1 expression (≥ 2 staining intensity in ≥ 5% of tumor cells) was 46% in MpBC, 6% in ER-positive cases, 6% in HER2-positive and 9% in TNBC [26]. Another American study comprising 27 cases of MpBC reported proportions of immune cell PD-L1 and tumor cell PD-L1 of 60% and 30%, respectively [44]. Finally, two smaller studies (*n* = 14 and 5) reported immune cell PD-L1-positivity in 50% and 80%, respectively, with a proportion of tumor cell PD-L1 of 40% positivity reported in the latter study [45, 46]. Thus, like in the present study, a substantial proportion of MpBCs seem to express PD-L1 both in immune cells and tumor cells. Interestingly, we found PD-L1 expression to vary among the different subtypes of MpBC with the highest expression of immune cell PD-L1 in squamous cell MpBC and the lowest in the mesenchymal MpBC subtype, a finding supported also by Lien et al. [43]. The heterogeneity of PD-L1 expression in subtypes of MpBC may partly explain some of the variation in results between studies.

The mean and median TIL counts in the present study were 9% and 5% for stromal TIL and 2% and 0 for intratumoral TIL, respectively. The counts in our work were lower compared to a recent meta-analysis of patients with TNBC where mean and median stromal TILs were 23.21% and 15%, respectively, and the corresponding intratumoral TILs 5.29% and 1% [24]. We found that TIL varied significantly between subtypes of MpBC. Like with PD-L1, the highest values of stromal TIL counts were found in squamous carcinomas, and the lowest in the mesenchymal subtype. We have identified two previous studies of TILs in MpBC. The study by Lien et al. reported similar findings as we did. The overall positivity for high or intermediate (> 10%) stromal TIL was 34% compared to 24% in the present study (data not shown). Additionally, they found the highest rate of TIL-positivity in squamous carcinomas (50%) and the lowest in matrix-producing MpBC (14.3%) [43]. Tray et al. reported median TIL percentages of 40 (*n* = 9) and 20 (*n* = 11) in MpBC patients with high vs. low TMB [7].

In the present study none of the investigated biomarkers were significantly associated with outcome, which is not surprising due to the limited statistical power with moderate patient number. In some previous studies, a correlation between PD-L1 expression and a more favorable prognosis was seen [38], although, in a recent meta-analysis there was no significant association between PD-L1 expression and survival outcomes in patients with TNBC [47]. A small previous study reported that high PD-L1 expression was a significant predictor of poor survival outcomes in MpBC patients when tested as a continuous variable [45]. Unlike PD-L1, TIL is, however, already an established prognostic factor in TNBC. A pooled analysis of 9 prospective trials in TNBC (*n* = 2148) treated with anthracyclines with or without taxanes indicated that stromal TIL and intratumoral TIL counts were highly significantly correlated and associated with both disease recurrence and OS (HR 0.83 and 0.76 for each 15 increase of TIL) [24].

Chemotherapy remains the treatment of choice for MpBC even though responses are scarce and the prognosis is poor. In our previous study we showed that the median overall survival time with metastatic disease was as low as 3.4 months and of those who received palliative chemotherapy only 6% had partial response [2]. In this context, there is an urgent need to identify novel targets for treatment of this rare disease. Immune check-point blockade including PD-1/PD-L1 -inhibitors have recently shown activity in a wide range of cancers including breast cancer [48]. In breast cancer, clinical trials on monotherapy have demonstrated efficacy in TNBC [38]. This suggests that immunotherapy might be effective also in MpBC as most of the cases are triple negative. The results of two randomized trials, IMpassion130 [27] and KEYNOTE-355 [41], have shown that immune check-point blockade added to palliative chemotherapy prolonged overall and progression free survival (PFS) among patients, whose tumors expressed PD-L1. A third phase 3 combination study, the IMpassion131 trial, however, was negative [49]. In the neoadjuvant setting, 5 randomized trials adding PD-1/PD-L1 blockade to chemotherapy increased the pathological complete response (pCR) rate in TNBC patients, although in 2 of these studies the result was not statistically significant [50–54].

In metastatic MpBC, promising activity was observed in a patient receiving pembrolizumab in combination with nab-paclitaxel [55]. Based on that study, MpBC was added to the phase 2 DART trial where a cohort of 17 MpBC patients received ipilimumab and nivolumab. The study met its primary endpoint with an overall response rate of 12% [56].

Predictive factors for efficacy of PD1/PD-L1 inhibitor-therapy in TNBC have been extensively studied. Presently, probably the most promising predictive factor is the one of the targets of immune-check-point therapy itself, the PD-L1-ligand. In all the three above-mentioned clinical trials no effect of PD1/PD-L1 inhibitor-therapy was seen in the PD-L1-negative population. It is important to note, however, the differences in assessing PD-L1-positivity in these studies. In the main analysis of IMpassion130, PD-L1 status was based on expression in immune cells while in KEYNOTE-119 and -355 PD-L1-expression in both tumor and immune cells were combined. According to a biomarker sub-study of the IMpassion130 trial both tumor and immune cell PD-L1 expression were predictive for immune check point therapy benefit, but the predictive impact of immune cell expression was stronger, and in multivariate analyses tumor cell PD-L1 expression was no longer significant in addition to immune cell expression [40]. In contrast, in the neoadjuvant setting PD1/PD-L1 blockade seems to benefit, i.e., increase pCR rate regardless of PDL1-expression [53]. The reason why PD-L1 expression predict efficacy of immune check-point inhibition in advanced breast cancer but not in the early-stage is presently unclear. Also, the number of TILs, have been suggested as a marker of immunogenicity [18, 57]. This is not surprising, since a number of previous studies have, like the present study, shown a strong association between the expression of PD-L1 and the number of TILs [47]. In addition, genomic instability and a high TMB have been associated to sensitivity to cancer immunotherapy [17–19].

The current study is limited by a relatively small sample size and a retrospective nature. Another limitation is the use of TMAs instead of full tissue sections especially for the evaluation of PD-L1 expression, as previous research has demonstrated that PD-L1 expression within core biopsy material from one breast cancer patient may vary even 4-fould between fields of view [38]. Considerable intratumoral heterogeneity in PD-L1 expression of TNBC has also been demonstrated by Dill et al. [46]. On the other hand, MpBC is a rare disease, and only a few previous marker studies of comparable size have been published.

## Conclusions

In conclusion, the high levels of expression of PD-L1, as well as markers of genomic instability (p53, γH2AX and pRPA2) indicate that MpBC is potentially immunogenic and may be a target for immune checkpoint therapy. Expression of TILs on the other hand seemed to be low compared to TNBC in general. We found significant heterogeneity in the expression of predictive factors for immune checkpoint therapy, indicating that the spindle and squamous cell carcinoma subtypes may be more promising immunotherapy targets than MpBC with mesenchymal differentiation. More clinical trials are needed to find out, whether the high expression of markers of immunogenicity in MpBC or some of its subtypes can be translated into benefit of immune checkpoint therapy.

## Supplementary Information

**Additional file 1: Table S1.** Clinicopathologic characteristics and immunohistochemical expression of p53, tcPD-L1 and icPD-L1.

## Data Availability

The datasets used and/or analyzed during the current study are available from the corresponding author on reasonable request.

## Abbreviations

BCOS: Breast cancer specific overall survival
DFS: Disease free survival
EGFR: Epidermal growth factor receptor
ER: Estrogen receptor
HR: Hazard ratio
H&E: Hematoxylin and eosin-stained
LPBC: Lymphocyte-predominant breast cancer
MpBC: Metaplastic breast cancer
pCR: Pathological complete response
PD-L1: Programmed death-ligand 1
PD-1: Programmed cell death protein 1
PFS: Progression free survival
PR: Progesterone receptor
RPA: Replication protein A
pRPA2: Phosphorylated-RPA2
TIL: Tumor infiltrating lymphocyte
TMA: Tissue microarray
TMB: Tumor mutation burden
TNBC: Triple negative breast cancer
γH2AX: Gamma-H2AX
