# Supplementary material for: Markers associated with genomic instability, immunogenicity and immune therapy responsiveness in Metaplastic carcinoma of the breast: Expression of γH2AX, pRPA2, P53, PD-L1 and tumor infiltrating lymphocytes in 76 cases
Source: BMC Cancer. 2022 Dec 12;22:1298. doi: 10.1186/s12885-022-10408-7 (PMC9743555; doi:10.1186/s12885-022-10408-7)
Supplement: Supplementary file 1 — Additional file 1: Table S1. Clinicopathologic characteristics and immunohistochemical expression of p53, tcPD-L1 and icPD-L1. [file 12885_2022_10408_MOESM1_ESM.docx]

**Table S1** Clinicopathologic characteristics and immunohistochemical expression of p53, tcPD-L1 and icPD-L1

| Features |  | p53 >10%  n (%) | p53 <10%  n (%) | *P* | tcPD-L1 >1%  n (%) | tcPD-L1 <1 %  n (%) | icPD-L1 >1%  n (%) | icPD-L1 <1%  n (%) |
| --- | --- | --- | --- | --- | --- | --- | --- | --- |
| Age at diagnosis ^a^* | ≤ 50  > 50 | 3 (6)  47 (94) | 1 (4)  23 (96) | 0.835 | 3 (7)  40 (93) | 1 (3)  29 (97) | 4 (9)  41 (91) | 0 (0)  28 (100) |
| Grade ^a^ | 1  2  3 | 1 (2)  6 (12)  43 (86) | 1 (4)  4 (17)  19 (79) | 0.449 | 0 (0)  5 (12)  38 (88) | 2 (7)  5 (17)  23 (77) | 2 (4)  4 (9)  39 (87) | 0 (0)  6 (21)  22 (79) |
| Pathological tumor size ^a^* (mm) | ≤ 20  21-50  > 50 | 11 (22)  26 (52)  13 (26) | 9 (38)  11 (46)  4 (17) | 0.184 | 8 (19)  23 (54)  12 (28) | 12 (40)  13 (43)  5 (17) | 10 (22)  25 (56)  10 (22) | 10 (36)  11 (39)  7 (25) |
| Pathological nodal stage ^a^ | N0  N1  N2  N3 | 38 (84)  4 (9)  2 (4)  1 (2) | 18 (78)  3 (13)  0 (0)  2 (9) | 0.513 | 33 (81)  4 (10)  2 (5)  2 (5) | 22 (85)  3 (12)  0 (0)  1 (4) | 35 (83)  4 (10)  1 (2)  2 (5) | 20 (80)  3 (12)  1 (4)  1 (4) |
| ER ^b^ | ≥ 10%  < 10% | 4 (8)  46 (92) | 19 (79)  5 (21) | 0.139 | 7 (16)  36 (84) | 2 (7)  28 (93) | 6 (13)  39 (87) | 3 (11)  25 (89) |
| PR ^b^ | ≥ 10%  < 10% | 1 (2)  49 (98) | 1 (4)  23 (96) | 0.546 | 0 (0)  43 (100) | 2 (7)  28 (93) | 1 (2)  44 (98) | 1 (4)  27 (96) |
| HER2 ^b^ | Positive  Negative | 3 (6)  47 (94) | 0 (0)  24 (100) | 0.546 | 2 (5)  41 (95) | 1 (3)  29 (97) | 2 (4)  43 (96) | 1 (4)  27 (96) |
| Ki-67 ^b^ | < 20  ≥ 20 | 6 (12)  44 (88) | 3 (13)  21 (88) | 1.000 | 6 (14)  37 (86) | 3 (10)  27 (90) | 5 (11)  40 (89) | 4 (14)  24 (86) |
| Basal phenotype ^c^ | Yes  No | 43 (86)  7 (14) | 18 (75)  6 (25) | 0.244 | 35 (81)  8 (19) | 25 (83)  5 (17) | 38 (84)  7 (16) | 22 (79)  6 (21) |
| gH2AX ^a^ | Median (range) | 41 (6 – 85) | 47 (5 – 85) | 0.882 | 39 (5 – 85) | 59 (8 – 83) | 38 (5 – 85) | 62 (6 – 85) |
| RPA2 ^a^ | Median (range) | 44 (3 – 81) | 42 (3 – 75) | 0.903 | 35 (3 – 81) | 55 (8 – 80) | 32 (3 – 81) | 52 (3 – 80) |
| sTIL ^a^ | Median (range) | 4 (0 – 60) | 5 (0 – 40) | 0.280 | 5 (0 – 60) | 3 (0 – 50) | 5 (0 – 60) | 1 (0 – 50) |
| iTIL ^a^ | Median (range) | 1 (0 – 20) | 0 (0 – 20) | 0.109 | 1 (0 – 20) | 0 (5 – 10) | 0 (0 – 20) | 0 (0 – 5) |
| tcPD-L1^a^ | Median (range) | 6 (0 – 100) | 3 (0 – 80) | 0.207 |  |  |  |  |
| icPD-L1^a^ | Median (range) | 20 (0 – 70) | 3 (0 – 80) | 0.165 |  |  |  |  |
| tcPD-L1 | >1% | 29 (60) | 20 (41) |  |  |  |  |  |
| icPD-L1 | >1% | 33 (67) | 12 (50) |  |  |  |  |  |

ER (estrogen receptor), PR (progesterone receptor), HER2 (human epidermal growth factor receptor 2), sTIL (stromal tumor-infiltrating lymphocytes), iTIL (intratumoral tumor-infiltrating lymphocytes), tcPD-L1 (tumor cell-PD-L1), icPD-L1 (immune cell PD-L1)

^a^ Mann-Whitney

^b^ Fisher’s exact test

^c^ χ2 test

* Age and tumor size tested as continuous variables
